# Sheffield General Infirmary

**Published:** 1892-11-12

**Authors:** 


### The Institutional Workshop.

### WITHIN THE HOSPITALS.

### SHEFFIELD GENERAL INFIRMARY.

By our Special Commissioner.

This is one of the oldest infirmaries in the country, and has been in existence nearly a century. It stands in the centre of the town, has ample open space all round it, but from the nature of the manufacturing business of Sheffield, the air is anything but salubrious. Ten years ago, perhaps even five, this institution would have presented few, if any, features of interest. It was behind the times, and many of the arrangements were opposed to all modern sanitary ideas. During the last five, and especially during the last three years, great and important changes have been made. The structural alterations alone must have cost a large sum, but the committee have the satisfaction of knowing that the buildings are now as complete as anything to be found at any reconstructed hospital, and the results of treatment are found to be eminently satisfactory. From one end of the hospital to the other, except in the new wing, perhaps, possibly some people may think, strange to say, everything was in excellent order. We noticed especially the good discipline in the wards, and the careful regulation of the ventilation, so that the atmosphere of all the wards was exceptionally good at the time of our visit between three and five o'clock in the afternoon. All this reflects the greatest credit on the resident medical and nursing staff, and we heartily congratulate Miss Rickards and Dr. Hugh Rhodes.

A Commendable Novelty.

In the board room are several portraits of worthies who have rendered good service to the institution, some of them being so well painted as to be pictures of value. Two of them represent old house surgeons. One of these portraits represents a gentleman apparently about seventy years of age, and we found on enquiry that he was for upwards of forty years house surgeon to the Sheffield General Infirmary. During that period he rendered exceptional service, took the deepest personal interest in the welfare of the institution, and came to regard it with as much affection as anything which absolutely belonged to himself. The other portrait is that of the house surgeon who succeeded the first, and remained for twenty-five years in continuous residence. It is not a little remarkable that any hospital should have been fortunate enough to have enjoyed such unselfish service at the hands of members of the profession, who, by this devotion, probably prevented themselves from securing much greater fame and wealth, which might have fallen to their lot had they followed the usual course. Hence, it is creditable to the governors of the institution which they represent that these two portraits should be suspended in the board-room of the Sheffield General Infirmary for the information and encouragement of all who come after. Another interesting fact in connection with the older portion of the buildings is the existence of a plan of the drainage at the time the foundations were laid, which shows that the architect was alive to the importance of keeping all the drains outside the buildings, and of disconnecting them from the common sewer by a ventilated manhole. We do not know the architect's name, we wish we did, as it is worthy of being permanently placed upon record as a remarkable example of a man who possessed intelligence at least fifty years in advance of his contemporaries. We hope the committee of the infirmary will take steps to have this plan carefully and permanently preserved.

The Infirmary Buildings.

These consist of two portions, the old or elder—that is, the main building, which is shaped like the letter L, and a well-proportioned and isolated pavilion. There is an excellent out-patient department at the back of the infirmary proper. Taking the pavilion block first, we would say that the four wards it contains are very pleasant, well-proportioned, and excellent types of the pavilion plan. It would be well, we think, if the committee were to recognize the fact that this building is distinct and apart from the main administration block, and if they were to make the salaries of the two Sisters who have charge of it slightly higher (at least £35 a-year) to emphasize the importance they attach to the extra responsibility necessarily thrown upon these two officers. They would thus secure as great efficiency in the management of these wards as undoubtedly prevails at the present time in the main building. The old buildings are remarkable, but satisfactory. They are remarkable in the sense that in each wing every ward opens into every other ward, and it would appear at first sight to be impossible to arrange to so reconstruct as to make them in such circumstances hygienically complete for the treatment of severe cases of disease. A careful inspection of the whole building leads us, however, to conclude, that this feat has been accomplished by the medical authorities of the Sheffield General Infirmary. The new lavatory and bath arrangements, occupying as they do remarkably little space, are exceedingly efficient. In fact, we commend them to the attention of architects and committees generally as types of what can be done in old buildings by the exercise of a little ingenuity and the application of experience and knowledge. There are three children's wards, one for boys between 11 and 14—a large ward, which was filled—one for boys under eleven years of age, also filled, and one for girls. It struck us as surprising that both at Bradford and Sheffield so many children, say sixty in each case, should be under treatment at the General Hospital, although each town possesses a Children's Hospital. It was pleasant to notice that the bedsteads throughout the institution were partly of brass, which, with the red coverlets, gave a wonderfully effective and bright appearance to all the wards. We were curious to know who attended to the brass and tin vessels, the brightness of which struck us as being exceptional. We were informed that the probationers did the work, and what was more, that they were proud of the appearance given to the wards by their labours in this direction. It seems to us that the way the great brass question is looked at, by most nurses, depends upon the amount of other work which devolves upon them. If there is a large enough staff, then a little polishing of the description referred to is not objected to, but rather the reverse.

### Special Fittings.

Sheffield has solved a difficulty which has exercised most hospital authorities. The managers have been enabled by an ingenious arrangement to provide a hydraulic passenger lift in each staircase. These lifts are relatively inexpensive, say £300 each, but they are most efficient and helpful to a large institution like that at Sheffield. There is evidence that considerable attention is paid to details throughout this infirmary. Each cot in the children's ward, for example, has its own little chair belonging to it. The toys are most tastefully arranged upon a shelf or table in the wards, forming a really attractive feature. In the lavatories, on the women's side, are fixed some wire racks, specially contrived, to enable Higginson's syringes to be properly cleansed and drained. These racks should be noted, as they ought to find their way into the lavatory of every well found hospital. Taking it altogether, although it is an old institution, we feel justified in declaring that the Sheffield General Infirmary, like the Salisbury Infirmary, is worthy of to-day, a fact which speaks well for all who are responsible for its administration. It is desirable that new lockers should be supplied throughout the whole of the wards, a fact which some wealthy manufacturer might make a note of, and if he would add to the note a measure of personal service by enquiring carefully at the Salisbury Infirmary, and one or two recent hospitals, which is the best type of locker, and then determine to supply 200 to the Sheffield Infirmary, he would render good service. If, 200 is thought to be too many for one manufacturer, then let there be a competition amongst the wealthy residents of Sheffield as to who shall do the work and let them divide the 200 lockers amongst themselves in any way they may prefer. The method is a matter of detail, but the lockers are a pressing necessity.

### The Nursing Staff.

Having said so much in praise of the many excellent points which this Infirmary presents, we must not pass over a grave defect. At the present time there is neither adequate nor proper, nay, not even reasonable accommodation for the nursing staff. In consequence, some of the wards have too few nurses for the work, and the whole institution, and the whole town of Sheffield is likely to suffer because of this. There is abundance of room on the present site to erect a nurses' home, large enough to provide accommodation for a staff adequate to supply all the needs of the Infirmary and those of the private citizens who desire to have trained nurses for the sick members of their families. In these circumstances we venture to hope that the people of Sheffield will come forward and second the Managing Committee in a determined endeavour to get this nurses' home erected without delay. If there should be any difficulty as to capital, then let the committee of the hospital guarantee the interest on the necessary capital sum, and make the interest and the sinking fund for the repayment of the capital a first charge on the profit earned by the private nursing staff. This has been done already in one or two cases, and if it is adopted by Sheffield, it will not only prove a financial success, but it will confer benefit upon the poor and also upon the better classes throughout Sheffield and the neighbourhood.

### The Resident Medical Staff.

Although there is a Medical School at Sheffield, there appears to be great difficulty in getting students to act as dressers, or to take an active and efficient part in the treatment of the in-patients. There are at present three resident medical officers upon whom devolves the whole duty of looking after the treatment of the patients who occupy the 200 beds, and their duties include that of taking cases and keeping the clinical records. As a matter of fact, it is far too much to expect any resident medical officer to undertake the duty of supplying the medical needs of 70 in-patients, unless he be provided with assistance in the shape of dressers and resident clinical clerks. We hope that the medical staff of the General Infirmary will confer with the Board, and that as a result steps will be taken to adopt the practice in force at the Leeds Infirmary, which secures a number of resident senior students, who act as dressers and clinical clerks under the resident medical officers. The sooner this course is adopted the better, as it will be not only for the benefit of the patients, but an act of justice to the resident medical officers.

